# Risk of hematologic malignancies following herpes zoster after COVID-19: a global cohort study

**DOI:** 10.3389/fmed.2025.1651614

**Published:** 2025-09-22

**Authors:** Chien-Lin Lu, Joshua Wang, Ching-Liang Ho, Yan-Jun Wu, Kuo-Cheng Lu, Chung-Chi Yang

**Affiliations:** ^1^Division of Nephrology, Department of Internal Medicine, Fu Jen Catholic University Hospital, Fu Jen Catholic University, New Taipei City, Taiwan; ^2^School of Medicine, College of Medicine, Fu Jen Catholic University, New Taipei City, Taiwan; ^3^Department of Research, Taipei Tzu Chi Hospital, Buddhist Tzu Chi Medical Foundation, New Taipei City, Taiwan; ^4^School of Biomedical Sciences, Queensland University of Technology, Brisbane, QLD, Australia; ^5^Division of Hematology and Oncology, Taipei Tzu Chi Hospital, Buddhist Tzu Chi Medical Foundation, New Taipei City, Taiwan; ^6^Department of Pediatrics, Taoyuan Armed Forces General Hospital, Taoyuan City, Taiwan; ^7^Division of Nephrology, Department of Medicine, Taipei Tzu Chi Hospital, Buddhist Tzu Chi Medical Foundation, New Taipei City, Taiwan; ^8^Division of Cardiovascular Medicine, Taoyuan Armed Forces General Hospital, Taoyuan, Taiwan; ^9^Division of Cardiovascular, Tri-Service General Hospital, National Defense Medical Center, Taipei, Taiwan; ^10^School of Medicine, National Tsing Hua University, Hsinchu, Taiwan; ^11^Institute of Bioinformatics and Structural Biology, National Tsing Hua University, Hsinchu, Taiwan

**Keywords:** acute leukemia, COVID-19 survivors, herpes zoster, leukopenia, multiple myeloma

## Abstract

**Introduction:**

Herpes zoster (HZ) has been reported as a potential post-viral complication in individuals recovering from COVID-19, possibly due to virus-induced immune dysregulation. We aimed to investigate whether post-COVID HZ is associated with an elevated risk of hematologic or infectious complications.

**Methods:**

We conducted a retrospective cohort study using the TriNetX global research network, which aggregates de-identified electronic health records from more than 140 healthcare institutions. Adults diagnosed with COVID-19 between January 2020 and January 2022 were stratified by the presence or absence of HZ within one year of infection and matched 1:1 by age, sex, and comorbidities. Outcomes including leukopenia, urinary tract infection, multiple myeloma, and acute leukemia were evaluated over a three-year follow-up using time-to-event and multivariable Cox regression analyses.

**Results:**

Individuals with post-COVID HZ had significantly higher risks of developing hematologic and infectious complications. Subgroup analyses identified older age, impaired kidney function, elevated inflammatory markers, and metabolic abnormalities as factors associated with greater risk.

**Discussion:**

These findings suggest that HZ following COVID-19 may serve as a clinical indicator of immune vulnerability and heightened susceptibility to hematologic and infectious disorders. Long-term monitoring may be warranted in high-risk populations.

## 1 Introduction

Herpes zoster (HZ), commonly known as shingles, results from the reactivation of latent varicella-zoster virus (VZV) in individuals who have previously contracted varicella. It is particularly prevalent among older adults and immunocompromised patients, whose cellular immunity is weakened by aging or underlying diseases (1). Since the emergence of the coronavirus disease 2019 (COVID-19) pandemic, growing evidence has suggested that individuals recovering from COVID-19 may be at elevated risk of HZ (2). This has been attributed to SARS-CoV-2-induced immune dysregulation, including lymphopenia, functional exhaustion of CD8 + T cells, and impaired interferon responses—all of which may compromise host control over latent viral infections (3). Several observational studies and case series have documented a temporal association between COVID-19 and increased HZ incidence (4, 5), further highlighting the need to understand the clinical implications of post-COVID HZ.

Beyond its acute dermatomal manifestations, HZ may serve as a clinical marker of underlying immune vulnerability and has been implicated in a range of systemic complications (6, 7). Prior studies have linked HZ to cardiovascular events, stroke, and long-term mortality, particularly in older or frail populations (8, 9). More recently, concerns have been raised regarding potential associations between HZ and hematologic malignancies such as multiple myeloma and acute leukemia (10, 11), as well as infectious complications including urinary tract infection (UTI) and leukopenia (12, 13). These associations are hypothesized to reflect chronic immune activation, bone marrow stress, or shared underlying risk factors.

In parallel, SARS-CoV-2 infection itself has been shown to induce persistent immune abnormalities long after the resolution of acute illness. These include prolonged T-cell dysfunction, altered cytokine profiles, dysregulated hematopoiesis, and changes in white blood cell lineages (14–16). Such disturbances may create a permissive environment for opportunistic infections and potentially promote malignant transformation. The interplay between COVID-19 and subsequent HZ may therefore signify compounded immunologic stress, amplifying vulnerability to downstream complications.

Despite these mechanistic insights, prior studies evaluating HZ-related outcomes have been limited by small sample sizes, single-center designs, or short follow-up periods (10, 11, 17). No large-scale, population-based study has yet comprehensively assessed whether individuals who develop HZ following COVID-19 are at increased risk for hematologic or infectious complications. In particular, the long-term risks of leukopenia, UTI, multiple myeloma, and acute leukemia in this population remain poorly defined.

To address this critical gap, we conducted a global cohort study using a large federated electronic health record platform to evaluate the risk of hematologic malignancies and immunologic complications among COVID-19 survivors with and without subsequent HZ. Leveraging robust propensity score matching (PSM) and three-year follow-up data, we aimed to determine whether HZ after COVID-19 serves as a benign reactivation event or a sentinel marker of deeper immune vulnerability. Our findings may provide important insights into risk stratification and guide post-COVID surveillance strategies for patients at heightened immunologic risk.

## 2 Materials and methods

### 2.1 Study design and data source

This retrospective, multicenter cohort study utilized the TriNetX Analytics Network, a federated global health research platform aggregating anonymized electronic health records (EHRs) from over 140 healthcare organizations worldwide. The platform captures structured data elements including demographics, diagnoses, medications, procedures, laboratory values, and vital status. Only deidentified analysis summaries of patient data can be accessed by users, ensuring that TriNetX operates in accordance with the Health Insurance Portability and Accountability Act (HIPAA) and General Data Protection Regulation (GDPR). Given the retrospective and anonymized nature of the data, the Institutional Review Board of Taipei Tzu Chi Hospital approved the study protocol with a waiver of informed consent (IRB No. 14-IRB043). The study was conducted in accordance with the Strengthening the Reporting of Observational Studies in Epidemiology (STROBE) guidelines. Following optimization, the final round of data analysis was performed on 18th May 2025.

### 2.2 Patient cohort and exposure classification

This study included adult patients (aged ≥18 years) with a confirmed diagnosis of COVID-19 recorded between January 1, 2020, and January 31, 2022. COVID-19 cases were identified through either a positive SARS-CoV-2 nucleic acid amplification test (TriNetX code: TNX: 9088) or a documented diagnosis using the ICD-10-CM code U07.1. Patients were subsequently classified into two exposure groups based on the occurrence of HZ following their COVID-19 diagnosis. The HZ cohort included individuals who received an HZ diagnosis within one year of their initial COVID-19 event, identified using ICD-10-CM codes B02 and its subcategories (B02.1–B02.9). Patients with any prior HZ diagnosis before COVID-19 or beyond the one-year window were excluded to ensure appropriate temporal sequencing.

**Figure 1:**
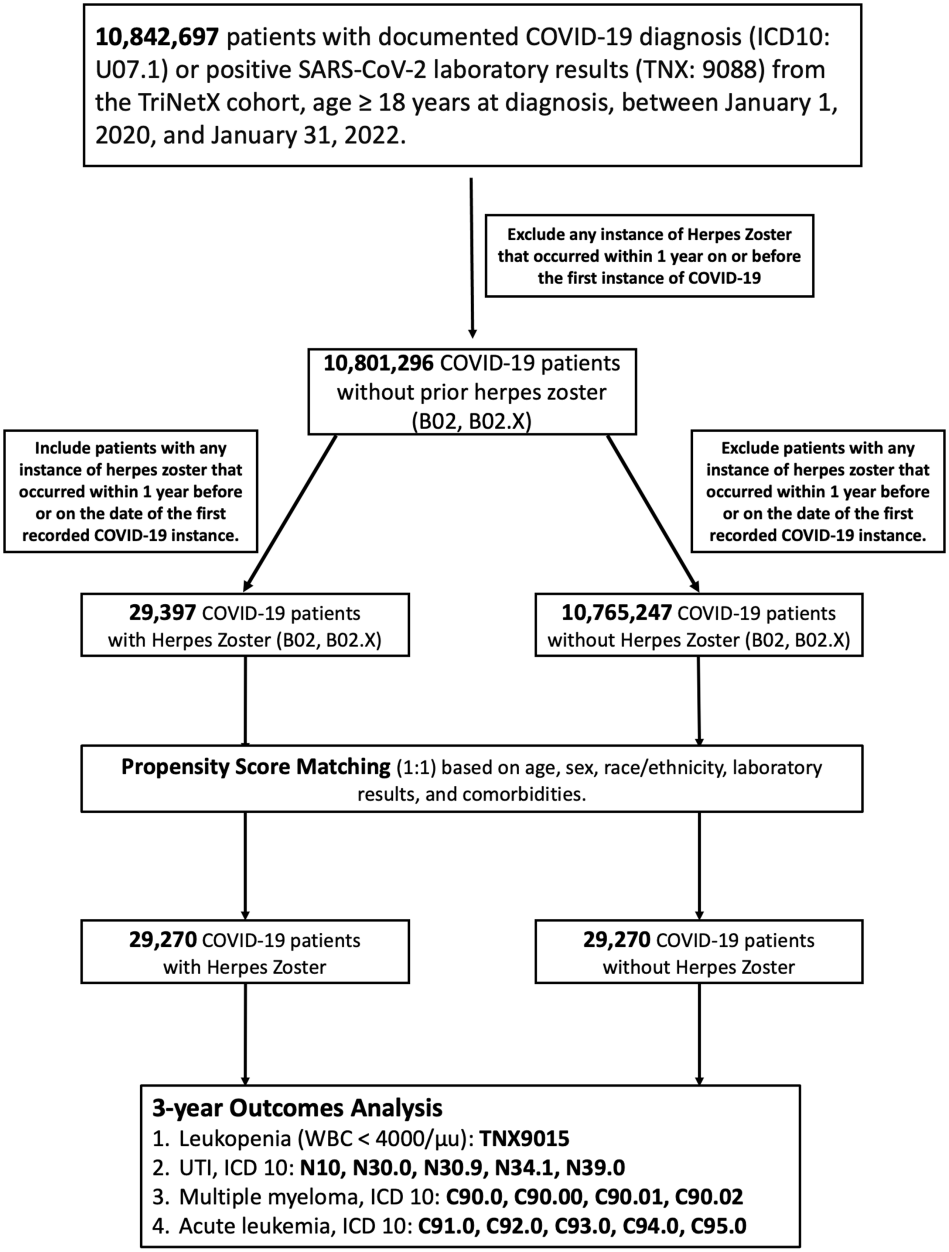
Cohort selection flowchart and outcome definitions. Flowchart illustrating the selection and matching of adult COVID-19 patients with and without HZ from the TriNetX network. A total of 29,270 patients with HZ following COVID-19 were matched 1:1 to controls without HZ. Four clinical outcomes were assessed over a 3-year follow-up: leukopenia, UTI, multiple myeloma, and acute leukemia. Abbreviations: COVID-19, Coronavirus Disease 2019; HZ, Herpes Zoster; ICD, International Classification of Diseases; UTI, urinary tract infection; WBC, white blood cell count.

After initial screening, 29,397 patients with post-COVID-19 HZ were identified. These were compared against a pool of 10,765,247 COVID-19 patients who had no HZ diagnosis during the observation period. Propensity score matching was later applied to generate two demographically and clinically comparable groups, each comprising 29,270 individuals. A detailed overview of the cohort selection process and outcome definitions is provided in Figure 1.

### 2.3 Index date and follow-up duration

The index date for each patient was defined as the date of the first COVID-19 diagnosis or the earliest positive SARS-CoV-2 test result, whichever occurred first. Follow-up began on the day after the index date and continued for a maximum of 1,095 days (equivalent to 3 years), until death, or until the last recorded clinical encounter, depending on which came first. Events that occurred on or prior to the index date were excluded from subsequent risk and time-to-event analyses to ensure that only new, post-COVID outcomes were captured.

### 2.4 Propensity score matching

To reduce baseline heterogeneity and minimize confounding, 1:1 PSM was performed using a greedy nearest-neighbor algorithm without replacement. The matching procedure incorporated a comprehensive set of covariates, including patient demographics, comorbid conditions, medication use, and key laboratory parameters. While our primary analyses of clinical outcomes—including leukopenia, UTI, multiple myeloma, and acute leukemia—were conducted in the full cohort of patients aged ≥18 years (*n* = 29,397 for the HZ group), the TriNetX platform imposes computational constraints that preclude full-scale matching across such a large population. To address this, we generated an age-restricted matched cohort comprising patients aged 60–70 years, yielding two equally sized groups (*n* = 7,404 each).

The age-restricted matched cohort demonstrated well-balanced baseline characteristics, as evidenced by standardized mean differences (SMDs) < 0.1 across nearly all variables (Supplementary Table S1). Although all outcomes were analyzed using the full cohort, the matched subgroup served to validate the robustness and consistency of findings. As shown in Table 1, hazard ratios (HRs) for key outcomes were largely comparable between the full and matched populations.

**Table 1:** Three-year outcome risks in the full and age-restricted COVID-19 cohorts with or without herpes zoster.

By age group	Outcomes	Cohorts	Patients in cohort	Patients with outcome	Survival probability at end of time window	Hazard ratio^a^	95% CI	Log-Rank test *p* value
Age 60–70	Leukopenia	With prior COVID-19 and HZ	6,122	671	88.37%	1.543	(1.364, 1.745)	<0.001
		With prior COVID-19 only	6,478	410	92.19%			
Full cohort		With prior COVID-19 and HZ	24,632	2,366	89.67%	1.515	(1.418, 1.617)	<0.001
		With prior COVID-19 only	26,413	1,436	92.92%			
Age 60–70	UTI	With prior COVID-19 and HZ	5,855	668	87.53%	1.498	(1.326, 1.692)	<0.001
		With prior COVID-19 only	6,275	421	91.25%			
Full cohort		With prior COVID-19 and HZ	22,621	2,936	85.62%	1.532	(1.444, 1.625)	<0.001
		With prior COVID-19 only	24,716	1,764	90.09%			
Age 60–70	Multiple myeloma	With prior COVID-19 and HZ	7,330	33	99.50%	2.856	(1.408, 5.795)	0.002
		With prior COVID-19 only	7,369	10	99.50%			
Full cohort		With prior COVID-19 and HZ	29,003	120	99.54%	3.159	(2.139, 4.666)	<0.001
		With prior COVID-19 only	29,176	32	99.85%			
Age 60–70	Acute leukemia	With prior COVID-19 and HZ	7,358	15	99.78%	1.322	(0.594, 2.944)	0.492
		With prior COVID-19 only	7,380	10	99.84%			
Full cohort		With prior COVID-19 and HZ	29,071	70	99.74%	2.713	(1.680, 4.381)	<0.001
		With prior COVID-19 only	29,200	22	99.91%			

CI, Confidence Interval; COVID, Coronavirus Disease 2019; HZ, Herpes Zoster; HR, Hazard Ratio; UTI, Urinary Tract Infection.

a Hazard ratio was adjusted using age at index, sex, race.

Notably, the association between HZ and acute leukemia reached statistical significance in the full cohort (HR = 2.713, 95% CI: 1.680–4.381; *p* < 0.001) but not in the age-restricted matched cohort (HR = 1.322, 95% CI: 0.594–2.944; *p* = 0.492), possibly due to the smaller number of incident cases and reduced power in the latter group. These findings highlight the enhanced sensitivity of the full-cohort analysis while underscoring the internal validity supported by PSM.

### 2.5 Outcome definitions

The clinical endpoints analyzed in this study included leukopenia, UTI, multiple myeloma, and acute leukemia. All outcome definitions were based on standardized diagnostic or laboratory codes available in the TriNetX platform. Patients with a history of the respective outcomes prior to the index date were excluded to ensure proper temporal sequencing between exposure and outcome. Leukopenia was identified using the Logical Observation Identifiers Names and Codes (LOINC)-coded laboratory test TNX:9015, representing total leukocyte count in peripheral blood. Patients whose most recent leukocyte value fell below 4,000/μL were classified as having leukopenia. UTI was defined using ICD-10-CM codes for UTI (N39.0), acute pyelonephritis (N10), acute cystitis (N30.0), cystitis unspecified (N30.9), and nonspecific urethritis (N34.1). Multiple myeloma was defined using the ICD-10-CM C90.0 series, which includes codes for active disease (C90.00), remission (C90.01), and relapse (C90.02). Acute leukemia encompassed a range of subtypes defined by ICD-10-CM codes, including acute lymphoblastic leukemia (C91.0), acute myeloblastic leukemia (C92.0), acute monoblastic or monocytic leukemia (C93.0), acute erythroid leukemia (C94.0), and unspecified acute leukemia (C95.0).

### 2.6 Sensitivity analysis

To ensure the robustness of our findings and account for potential confounding from pre-existing immunosuppressive conditions, we conducted a sensitivity analysis by excluding patients with documented immunosuppression within 1 year prior to their COVID-19 diagnosis. Immunosuppression was defined using ICD-10-CM codes indicative of long-term immunosuppressive therapy or immune compromise, including: Z79.62 (long-term use of immunosuppressants), Z79.899 (other long-term drug therapy), D84.821 (immunodeficiency due to drugs), D89.8 (other specified immune mechanism disorders), and D90 (immune compromise due to radiation or chemotherapy). Patients meeting any of these criteria were excluded from the sensitivity cohort. We then reanalyzed the three-year risks of leukopenia, UTI, multiple myeloma, and acute leukemia using the same analytical framework as the primary analysis, including Kaplan–Meier survival estimates and Cox proportional hazards modeling. This approach allowed us to assess whether the observed associations persisted in a population without baseline immune suppression.

### 2.7 Statistical analyses

Outcome comparisons were conducted using both risk-based and time-to-event analytic approaches. Risk metrics including absolute risk, risk differences, risk ratios (RR), and odds ratios (ORs) were estimated using the TriNetX risk analysis module, excluding patients with a prior diagnosis of the outcome. Kaplan–Meier survival analyses were performed for time-to-event endpoints, and intergroup differences were assessed using log-rank tests. Cox proportional hazards models were used to compute HRs with 95% confidence intervals (CIs). All statistical procedures were performed within the TriNetX cloud-based environment. A two-tailed *p*-value <0.05 was considered statistically significant.

## 3 Results

### 3.1 Leukopenia (WBC < 4,000/μL)

**Figure 2:**
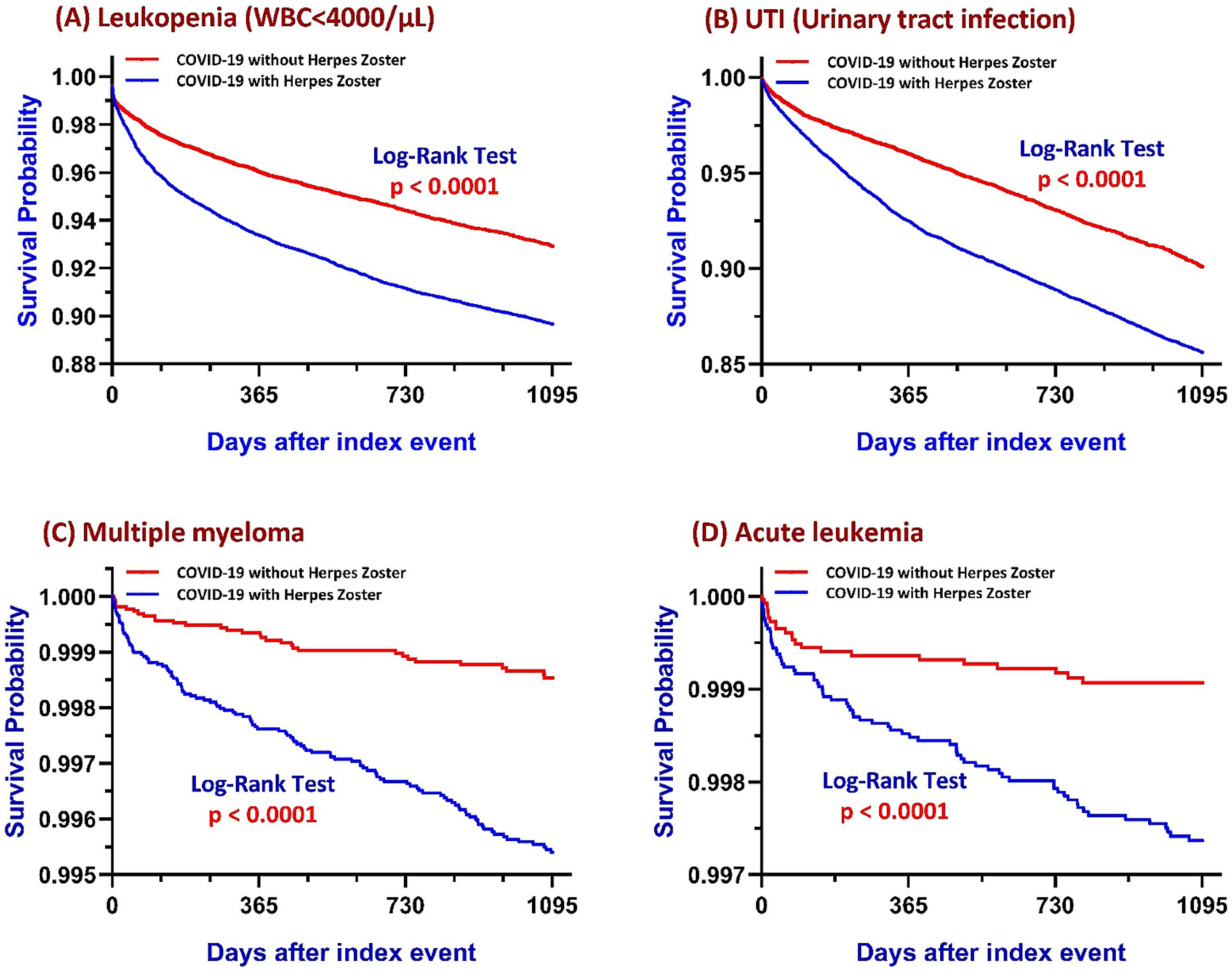
Kaplan–Meier survival curves comparing COVID-19 survivors with and without HZ over a three-year follow-up period for four outcomes: **(A)** Leukopenia (WBC < 4,000/μL), **(B)** UTI, **(C)** Multiple myeloma, and **(D)** Acute leukemia. In each panel, the blue line represents patients with HZ and the red line represents those without HZ. Log-rank tests were used to compare survival distributions between groups, with all panels demonstrating statistically significant differences (*p* < 0.0001). Abbreviations: COVID-19, coronavirus disease 2019; HZ, herpes zoster; WBC, white blood cell count; UTI, urinary tract infection.

HZ patients had a higher incidence of leukopenia (3.585% vs. 2.185%), corresponding to an absolute risk difference of 1.400% (95% CI: 1.093–1.707%), a RR of 1.641, and an OR of 1.671 (95% CI: 1.497–1.866; *p* < 0.001). Kaplan–Meier survival analysis further confirmed significantly lower leukopenia-free survival in the HZ group (log-rank *p* < 0.001), with a HR of 1.515 (95% CI: 1.418–1.617), indicating a 51.5% increased hazard of developing leukopenia over the follow-up period (Figure 2A).

**Figure 3:**
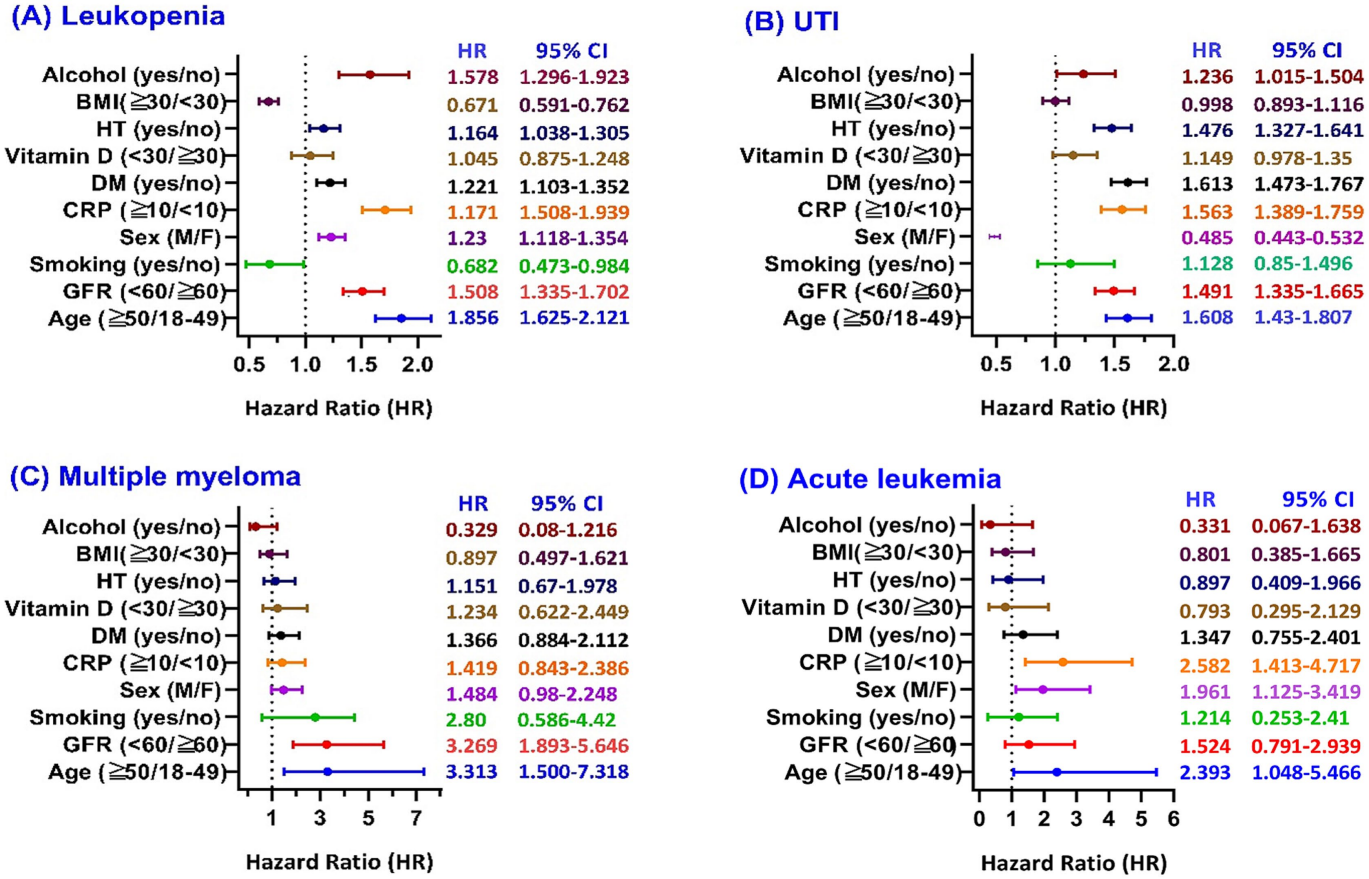
Subgroup analyses of risk factors associated with post-COVID HZ–related complications. Forest plots display HRs and 95% confidence intervals (CIs) for major co-variates across four outcomes: **(A)** Leukopenia (WBC < 4,000/μL), **(B)** UTI, **(C)** Multiple myeloma, and **(D)** Acute leukemia. Abbreviations: BMI, body mass index; CRP, C-reactive protein; DM, diabetes mellitus; GFR, glomerular filtration rate; HT, hypertension; UTI, urinary tract infection.

Subgroup analysis (Figure 3A) revealed significantly elevated leukopenia risk among patients with age ≥50 years (HR: 1.856), impaired renal function (GFR < 60; HR: 1.508), male sex (HR: 1.230), elevated C-reactive protein (CRP) ≥ 10 mg/L (HR: 1.710), diabetes mellitus (HR: 1.221), hypertension (HR: 1.164), and alcohol use (HR: 1.578). In contrast, smoking (HR: 0.682) and obesity (BMI ≥ 30; HR: 0.671) were associated with significantly lower leukopenia risk. No statistically significant difference was observed in the subgroup defined by vitamin D status.

### 3.2 Urinary tract infection

Patients with HZ following COVID-19 exhibited a significantly higher incidence of UTIs compared to COVID-19 survivors without HZ (13.0% vs. 7.1%), corresponding to an absolute risk difference of 5.8% (95% CI: 5.3–6.4%). The RR was 1.819, and the OR was 1.941 (95% CI: 1.824–2.065; *p* < 0.001). Kaplan–Meier survival analysis demonstrated a significantly lower UTI-free survival probability in the HZ group (log-rank *p* < 0.001), with a HR of 1.532 (95% CI: 1.444–1.625), indicating a persistently elevated risk of UTI throughout the follow-up period (Figure 2B).

Subgroup analysis (Figure 3B) showed that the risk of UTI was significantly increased in patients aged ≥50 years (HR: 1.608), with impaired renal function (GFR < 60; HR: 1.491), elevated CRP levels (≥10 mg/L; HR: 1.563), diabetes mellitus (HR: 1.613), hypertension (HR: 1.476), and alcohol use (HR: 1.236). Conversely, male sex was associated with a significantly reduced UTI risk (HR: 0.485). No significant association was observed in subgroups defined by smoking status, vitamin D status, or BMI.

### 3.3 Multiple myeloma

In the matched cohort, individuals who developed HZ after recovering from COVID-19 exhibited a significantly higher incidence of multiple myeloma compared to those who had COVID-19 without subsequent HZ (0.4% vs. 0.1%). The absolute risk difference was 0.3% (95% CI: 0.2–0.4%), with a RR of 3.772 and an OR of 3.784 (95% CI: 2.561–5.590; *p* < 0.001). Kaplan–Meier survival analysis further confirmed a significantly lower myeloma-free survival in the post-COVID HZ group (log-rank *p* < 0.001), with a HR of 3.159 (95% CI: 2.139–4.666), indicating a sustained and substantially elevated risk over time (Figure 2C).

Subgroup analysis (Figure 3C) demonstrated significantly elevated multiple myeloma risk in patients aged ≥50 years (HR: 3.313) and those with impaired renal function (GFR < 60; HR: 3.269). No statistically significant differences were observed across subgroups defined by smoking status, sex, CRP levels, diabetes, vitamin D status, hypertension, BMI, or alcohol use.

### 3.4 Acute leukemia

The risk of acute leukemia was significantly elevated among patients who developed HZ after COVID-19, with an incidence of 0.24% (70 cases), compared to 0.08% (22 cases) in those without HZ. This corresponded to an absolute risk difference of 0.2% (95% CI: 0.1–0.2%), a RR of 3.196, and an OR of 3.201 (95% CI: 1.982–5.170; *p* < 0.001). Kaplan–Meier survival analysis demonstrated a clear divergence in leukemia-free survival curves between groups (log-rank *p* < 0.001), with a HR of 2.713 (95% CI: 1.680–4.381), indicating a sustained increase in instantaneous risk over the three-year follow-up period (Figure 2D).

Subgroup analysis (Figure 3D) revealed significantly increased acute leukemia risk in patients aged ≥50 years (HR: 2.393), males (HR: 1.961), and those with elevated CRP ≥ 10 mg/L (HR: 2.582). No statistically significant associations were observed across subgroups defined by renal function, smoking status, diabetes mellitus, vitamin D levels, hypertension, BMI, or alcohol use.

### 3.5 Chronic leukemia and lymphoma

The incidence of chronic leukemia was higher in patients with HZ (83 cases among 30,758 individuals) than in those without HZ (46 cases among 30,903 individuals), yielding a HZ of 1.537 (95% CI: 1.072–2.204; *p* = 0.018). Similarly, the risk of lymphoma was elevated in the HZ group, with 215 cases among 30,323 individuals versus 89 cases among 30,761 individuals in the non-HZ group (HR: 2.079, 95% CI: 1.624–2.662; *p* < 0.001) (Supplementary Table S2).

### 3.6 Healthcare utilization following COVID-19

To further characterize healthcare utilization patterns, we analyzed the distribution and frequency of outpatient and inpatient encounters in both cohorts (Supplementary Table S3). Among patients who developed HZ following COVID-19, 92.7% (28,770/31,015) had outpatient visits, and 38.9% (12,066/31,015) had inpatient encounters. In contrast, in the COVID-19 without HZ cohort, 78.8% (24,439/31,015) had outpatient visits, and 28.6% (8,883/31,015) had inpatient encounters. The mean number of outpatient visits was significantly higher in the HZ group compared to the non-HZ group (43.94 ± 52.52 vs. 23.96 ± 38.60; *p* < 0.001). Similarly, the mean number of inpatient visits was also elevated in the HZ group (2.21 ± 6.62 vs. 1.21 ± 4.06; p < 0.001). These findings suggest a higher burden of healthcare utilization among COVID-19 survivors who subsequently developed HZ.

### 3.7 Sensitivity analysis

In the sensitivity analysis restricted to patients without immunosuppression prior to COVID-19, the associations between HZ and adverse clinical outcomes remained statistically significant and directionally consistent with the main findings. Among immunocompetent individuals, HZ following COVID-19 was associated with significantly increased three-year risks of leukopenia (HR = 1.682, 95% CI: 1.543–1.833), UTI (HR = 1.667, 95% CI: 1.545–1.799), multiple myeloma (HR = 2.309, 95% CI: 1.476–3.613), and acute leukemia (HR = 2.882, 95% CI: 1.554–5.343), with all log-rank *p*-values < 0.001. These results suggest that the elevated risks observed in the overall cohort were not driven by baseline immunosuppressive conditions and underscore a robust independent association between post-COVID HZ and subsequent hematologic and infectious complications. Detailed results are presented in Table 2.

**Table 2:** Sensitivity analysis excluding patients with preexisting immunosuppression prior to COVID-19.

Exclude immuno-suppression*	Outcomes	Cohorts	Patients in cohort	Patients with outcome	Survival probability at end of time window	Hazard ratio^a^	95% CI	Log-Rank test *p* value
Yes	Leukopenia	With prior COVID-19 and HZ	16,926	1,495	90.41%	1.682	(1.543, 1.833)	<0.001
		With prior COVID-19 only	17,924	792	94.03%			
No		With prior COVID-19 and HZ	24,632	2,366	89.67%	1.515	(1.418, 1.617)	<0.001
		With prior COVID-19 only	26,413	1,436	92.92%			
Yes	UTI	With prior COVID-19 and HZ	15,633	1,891	86.52%	1.667	(1.545, 1.799)	<0.001
		With prior COVID-19 only	16,925	1,016	91.46%			
No		With prior COVID-19 and HZ	22,621	2,936	85.62%	1.532	(1.444, 1.625)	<0.001
		With prior COVID-19 only	24,716	1,764	90.09%			
Yes	Multiple myeloma	With prior COVID-19 and HZ	19,317	73	99.58%	2.309	(1.476, 3.613)	<0.001
		With prior COVID-19 only	19,387	26	99.80%			
No		With prior COVID-19 and HZ	29,003	120	99.54%	3.159	(2.139, 4.666)	<0.001
		With prior COVID-19 only	29,176	32	99.85%			
Yes	Acute leukemia	With prior COVID-19 and HZ	19,343	45	99.74%	2.882	(1.554, 5.343)	<0.001
		With prior COVID-19 only	19,411	13	99.91%			
No		With prior COVID-19 and HZ	29,071	70	99.74%	2.713	(1.680, 4.381)	<0.001
		With prior COVID-19 only	29,200	22	99.91%			

CI, confidence interval; COVID-19, coronavirus disease 2019; HZ, herpes zoster; HR, hazard ratio; ICD-10-CM, International Classification of Diseases, 10th Revision, Clinical Modification; UTI, urinary tract infection; WBC, white blood cell.

a Hazard ratio was adjusted using age at index, sex, race.

## 4 Discussion

In this large-scale, propensity score–matched, multinational cohort study, we found that individuals who developed HZ after recovering from COVID-19 faced significantly elevated risks of leukopenia, UTI, multiple myeloma, and acute leukemia over a three-year follow-up period, compared to matched controls without HZ. Additionally, the risks of chronic leukemia and lymphoma were also significantly increased in patients with HZ. However, our study followed patients for a maximum of 3 years after the index date, which may not be sufficient to fully capture the long-term risk of chronic hematological malignancies. Chronic leukemia and lymphoma often have prolonged latency periods, and longer follow-up would be necessary to more accurately assess their association with HZ (18–22). These associations remained robust after adjustment for demographic factors, comorbidities, medication use, and laboratory parameters, suggesting that HZ may reflect not only viral reactivation but also signal an underlying state of immunologic vulnerability. Notably, most HZ diagnoses in our study were made in the ambulatory care setting, consistent with the real-world observation that HZ is typically managed without hospitalization. This supports the generalizability of our findings to outpatient clinical practice.

Subgroup analyses further revealed that certain clinical characteristics may amplify these risks. For both leukopenia and UTI, higher risk was observed among individuals aged ≥50 years, those with impaired renal function, elevated CRP levels, diabetes mellitus, hypertension, and alcohol use. Interestingly, smoking and obesity were associated with significantly lower leukopenia risk, while male sex conferred a reduced risk of UTI, highlighting potential sex- and metabolism-related differences in immune susceptibility. In contrast, the heightened risks of multiple myeloma and acute leukemia were most prominent among older adults, individuals with renal impairment, and those with elevated inflammatory markers, suggesting a possible link to hematopoietic fragility or subclinical clonal evolution under post-viral immune pressure. Taken together, these findings underscore the importance of recognizing HZ following COVID-19 as a clinically meaningful signal of immunologic and hematologic risk, warranting proactive surveillance and targeted follow-up.

HZ is well established as a clinical indicator of impaired cell-mediated immunity, particularly among aging or immunocompromised individuals (1, 7, 23). In the context of prior COVID-19, this immune vulnerability may be amplified through mechanisms such as lymphopenia, CD8 + T-cell exhaustion, and interferon pathway suppression (24–27). These combined immune insults may impair both antiviral control and immunologic surveillance of latent infections and malignant precursors. Our observed associations with leukopenia and UTI further support this hypothesis, suggesting that HZ may not simply reflect viral reactivation, but may also represent a warning signal of systemic immunologic fragility.

Leukopenia, which affected 3.6% of individuals in the HZ group compared to 2.2% in the control group, along with a 1.5-fold increase in UTI risk, may serve as actionable markers of compromised host defenses. These complications likely reflect disturbances in myelopoiesis, increased infection susceptibility, and dysregulated innate immunity (28–30). The risks were notably heightened in patients with renal impairment, elevated inflammatory markers, and metabolic comorbidities—all of which are known to exacerbate immune senescence and suppress hematopoietic resilience (31, 32). These findings emphasize the utility of leukopenia and UTI as indicators of broader immunocompromised among post-COVID patients with HZ.

The associations observed between HZ and subsequent multiple myeloma and acute leukemia suggest that ongoing immune dysfunction may not only predispose individuals to infection but also facilitate malignant transformation (11, 17). Chronic immune stimulation—whether due to viral reactivation, unresolved inflammation, or apoptotic failure—can promote clonal hematopoiesis and genomic instability (33–36). In multiple myeloma, inflammatory cytokines such as IL-6 and TNF-*α* have been shown to drive plasma cell proliferation and survival (37–40). Our study demonstrated a nearly threefold increase in multiple myeloma risk among HZ patients, particularly in older adults, smokers, and those with chronic kidney disease, all of whom are recognized as high-risk populations for myeloma development.

These results are consistent with prior literature showing that multiple myeloma patients during the COVID-19 pandemic experienced higher infection rates, disrupted care, and reduced survival (41–43). Additionally, HZ has previously been linked to increased hematologic malignancy risk, possibly via sustained immunologic perturbation (10, 11, 44). A notable case even described spontaneous multiple myeloma remission following SARS-CoV-2 infection, suggesting that COVID-19–induced immune alterations may exert paradoxical effects on malignant clones (45).

For acute leukemia, our data revealed a 2.7-fold higher incidence following HZ in COVID-19 survivors. Historical and modern studies have suggested that HZ may act as a prodrome or unmasking event in the pathogenesis of leukemia, particularly in immunologically vulnerable individuals (46). The inflammatory and hematopoietic stress induced by SARS-CoV-2 infection could further exacerbate these risks. Prior research has identified post-COVID perturbations in leukocyte populations, including monocytosis and thrombocytosis, potentially representing early signs of dysregulated hematopoiesis (47, 48). These immune shifts, compounded by HZ-related immune stress, may unmask subclinical clonal evolution in susceptible individuals.

In clinical practice, the development of herpes zoster following COVID-19 should raise concern for possible underlying hematologic disorders. If patients present with persistent low blood cell counts, unusual patterns of zoster rash, or unexplained systemic inflammatory symptoms, further hematologic evaluation may be warranted—such as complete blood counts, bone marrow biopsy, or molecular genetic testing to investigate potential clonal hematopoiesis or early-stage malignancy.

Several limitations should be acknowledged in interpreting our findings. First, this was a retrospective cohort study based on electronic health records from the TriNetX global network, which, although extensive, relies on the accuracy and completeness of coding practices across diverse healthcare settings. Misclassification of herpes zoster or outcome diagnoses cannot be entirely excluded. Second, while we performed rigorous PSM and sensitivity analyses, residual confounding may still be present due to unmeasured variables such as socioeconomic status, over-the-counter medication use, vaccination history (e.g., zoster or COVID-19 vaccines), and genetic predispositions. Third, the temporal association between HZ and subsequent complications suggests a potential risk signal but does not establish causality. It remains possible that HZ acts as a clinical marker of latent immunologic or malignant processes already underway, rather than a direct contributor to disease pathogenesis. Fourth, although our large unmatched cohort enhanced statistical power, the age-restricted matched cohort (60–70 years) may have limited generalizability to younger or older populations, especially for rarer outcomes like acute leukemia. Fifth, the time-to-event analyses used in this study censor patients whose last clinical fact was recorded during the observation period. Given that our outcome period ends in January 2025 at the latest, a considerable number of patients may have been excluded from the time-to-event analyses, potentially skewing the results if censoring was not evenly distributed between cohorts. This limitation was particularly evident in the prevalence differences for leukopenia between the risk-based and time-to-event analyses. Nonetheless, outcome prevalence remained consistent across methods for all other measured outcomes. Sixth, due to platform constraints, leukopenia could only be identified through a single laboratory value—specifically, the most recent WBC count during the outcome window. This definition may miss patients whose leukopenia resolved through clinical intervention prior to their most recent test, leading to an underestimation of true prevalence. However, since this limitation applies equally to both cohorts, it is unlikely to introduce significant bias into the estimated risk ratios. Lastly, although our findings were robust across multiple analytic approaches and sensitivity tests, future studies incorporating longitudinal laboratory trajectories, detailed vaccination records, and functional immune profiling are warranted to clarify the mechanistic pathways linking post-COVID HZ to hematologic and infectious complications.

Despite these constraints, our study provides compelling evidence that HZ following COVID-19 may represent a high-risk immunologic phenotype with implications for infection susceptibility and malignant transformation. Future mechanistic investigations into post-viral immune remodeling, clonal hematopoiesis, and epigenetic alterations may offer insights into the pathways linking HZ to hematologic complications.

## 5 Conclusion

HZ following COVID-19 is associated with elevated risks of both immunologic (leukopenia and UTI) and hematologic (multiple myeloma and acute leukemia) complications. Rather than being a benign post-viral occurrence, HZ may indicate deeper immunologic instability. Further mechanistic studies are warranted to explore how viral reactivation, immune exhaustion, and clonal evolution intersect in the post-COVID setting. Improved longitudinal monitoring, including immune profiling and genomic surveillance, may facilitate early detection and intervention in patients at highest risk.

## Data Availability

The data analyzed in this study is subject to the following licenses/restrictions: due to licensing and privacy restrictions, the data used in this study from the TriNetX Global Health Research Network are not publicly available. TriNetX provides access to de-identified, aggregate-level data obtained from a global network of healthcare organizations. Researchers may request access to the dataset used in this study through the TriNetX website (https://trinetx.com) or by contacting Privacy@TriNetX.com. Data are available on reasonable request from the corresponding author.

## References

[1] McKay SL Guo A Pergam SA Dooling K. Herpes zoster risk in immunocompromised adults in the United States: a systematic review. Clin Infect Dis. (2020) 71:e125–34. doi: 10.1093/cid/ciz1090, PMID: 31677266 PMC7195255

[2] Chen YC Ho CH Liu TH Wu JY Huang PY Tsai YW. Long-term risk of herpes zoster following COVID-19: a retrospective cohort study of 2 442 686 patients. J Med Virol. (2023) 95:e28745. doi: 10.1002/jmv.28745, PMID: 37431965

[3] Mishra KP Singh M Saraswat D Ganju L Varshney R. Dysfunctional state of T cells or exhaustion during chronic viral infections and COVID-19: a review. Viral Immunol. (2022) 35:284–90. doi: 10.1089/vim.2022.0002, PMID: 35325564

[4] Parikh R Yousefi M Curran D Widenmaier R. The impact of the COVID-19 pandemic on the incidence of herpes zoster: a narrative literature review. Infect Dis Ther. (2024) 13:447–61. doi: 10.1007/s40121-024-00924-3, PMID: 38441844 PMC10965887

[5] Narasimhan M Ramakrishnan R Durai PCT Sneha B. Association between COVID-19 infection and herpes zoster: a case series. J Family Med Prim Care. (2023) 12:2516–9. doi: 10.4103/jfmpc.jfmpc_2112_22, PMID: 38074274 PMC10706513

[6] Staikov I Neykov N Marinovic B Lipozenčić J Tsankov N. Herpes zoster as a systemic disease. Clin Dermatol. (2014) 32:424–9. doi: 10.1016/j.clindermatol.2013.11.010, PMID: 24767191

[7] Soyuncu S Berk Y Eken C Gulen B Oktay C. Herpes zoster as a useful clinical marker of underlying cell-mediated immune disorders. Ann Acad Med Singap. (2009) 38:136–8. doi: 10.47102/annals-acadmedsg.V38N2p136, PMID: 19271041

[8] Warren-Gash C. Herpes zoster: epidemiological links with stroke and myocardial infarction. J Infect Dis. (2018) 218:S102–6. doi: 10.1093/infdis/jiy385, PMID: 30247593 PMC6904293

[9] Curhan SG Kawai K Yawn B Rexrode KM Rimm EB Curhan GC. Herpes zoster and long-term risk of cardiovascular disease. J Am Heart Assoc. (2022) 11:e027451. doi: 10.1161/JAHA.122.027451, PMID: 36382961 PMC9851464

[10] Kim M Han K Yoo SA Lee JH. Herpes zoster and subsequent cancer risk: a nationwide population-based cohort study in Korea. Dermatology. (2021) 237:73–8. doi: 10.1159/000505911, PMID: 32114571

[11] Mahale P Yanik EL Engels EA. Herpes zoster and risk of cancer in the elderly U.S. population. Cancer Epidemiol Biomarkers Prev. (2016) 25:28–35. doi: 10.1158/1055-9965.EPI-15-1033, PMID: 26578536 PMC4713252

[12] Seixas R Dias F Ribeiro A Sobral S Rita H. Herpes zoster infection in an immunocompromised patient: a case report and review of corticosteroid's role. Cureus. (2022) 14:e20908. doi: 10.7759/cureus.20908, PMID: 35004077 PMC8727328

[13] Almutairi N Almutairi AN Almazyad M Alwazzan S. Herpes zoster in the era of COVID 19: A prospective observational study to probe the association of herpes zoster with COVID 19 infection and vaccination. Dermatol Ther. (2022) 35:e15521. doi: 10.1111/dth.15521, PMID: 35434963 PMC9111648

[14] Lim J Puan KJ Wang LW Teng KWW Loh CY Tan KP. Data-driven analysis of COVID-19 reveals persistent immune abnormalities in convalescent severe individuals. Front Immunol. (2021) 12:710217. doi: 10.3389/fimmu.2021.710217, PMID: 34867943 PMC8640498

[15] Phetsouphanh C Darley DR Wilson DB Howe A Munier CML Patel SK. Immunological dysfunction persists for 8 months following initial mild-to-moderate SARS-CoV-2 infection. Nat Immunol. (2022) 23:210–6. doi: 10.1038/s41590-021-01113-x, PMID: 35027728

[16] Opsteen S Files JK Fram T Erdmann N. The role of immune activation and antigen persistence in acute and long COVID. J Investig Med. (2023) 71:545–62. doi: 10.1177/10815589231158041, PMID: 36879504 PMC9996119

[17] Iglar K Kopp A Glazier RH. Herpes zoster as a marker of underlying malignancy. Open Med. (2013) 7:e68–73. 24348886 PMC3863753

[18] Michiels J Kate F Raeve H Gadisseur A. Bone marrow features and natural history of BCR/ABL-positive thrombocythemia and chronic myeloid leukemia compared to BCR/ABL negative thrombocythemia in essential thrombocythemia and polycythemia vera. J Hematol Thromb Dis. (2015) 3:1–9. doi: 10.4172/2329-8790.1000192, PMID: 39887974

[19] Ekwere T Abudu E. BCR-ABL positive childhood chronic myeloid leukemia. J Case Rep. (2017) 7:289–92.

[20] Büyükpamukçu M Varan A Yazıcı N Akalan N Söylemezoğlu F Zorlu F. Second Malignant Neoplasms Following the Treatment of Brain Tumors in Children. J Child Neurol. (2006) 21:433–6. doi: 10.1177/08830738060210050901, PMID: 16901454

[21] Polychronopoulou S Panagiotou J Papadakis T Mavrou A Anagnostou D Haidas S. Secondary malignancies in a child with Hodgkin's disease: T-cell lymphoma and myelodysplastic syndrome evolving into acute nonlymphoblastic leukaemia. Med Pediatr Oncol. (1996) 26:359–66. doi: 10.1002/(SICI)1096-911X(199605)26:5<359::AID-MPO9>3.0.CO;2-H, PMID: 8614370

[22] Keresztes K Miltényi Z András C Illés Á. Second malignancies in patients treated for Hodgkin's disease. Magy Onkol. (2002) 46:247–51. PMID: 12368920

[23] Oxman MN. Herpes zoster pathogenesis and cell-mediated immunity and immunosenescence. J Am Osteopath Assoc. (2009) 109:S13–7. 19553630

[24] Zhang S Asquith B Szydlo R Tregoning JS Pollock KM. Peripheral T cell lymphopenia in COVID-19: potential mechanisms and impact. Immunother Adv. (2021) 1:ltab 015. doi: 10.1093/immadv/ltab015, PMID: 35965490 PMC9364037

[25] Alharbi KS Singh Y Prasad Agrawal G Altowayan WM Almalki WH Sharma A. Synergism of CD28 immune molecule in late immunosuppressive phase of COVID-19: effectiveness in vaccinated individuals. Altern Ther Health Med. (2023) 29:67–73. 35212647

[26] Alahdal M Elkord E. Exhaustion and over-activation of immune cells in COVID-19: Challenges and therapeutic opportunities. Clin Immunol. (2022) 245:109177. doi: 10.1016/j.clim.2022.109177, PMID: 36356848 PMC9640209

[27] Roe K. A role for T-cell exhaustion in Long COVID-19 and severe outcomes for several categories of COVID-19 patients. J Neurosci Res. (2021) 99:2367–76. doi: 10.1002/jnr.24917, PMID: 34288064 PMC8427009

[28] Ing VW. The etiology and management of leukopenia. Can Fam Physician. (1984) 30:1835–9. PMID: 21279100 PMC2154209

[29] Neth OW Bajaj-Elliott M Turner MW Klein NJ. Susceptibility to infection in patients with neutropenia: the role of the innate immune system. Br J Haematol. (2005) 129:713–22. doi: 10.1111/j.1365-2141.2005.05462.x, PMID: 15952996

[30] Belok SH Bosch NA Klings ES Walkey AJ. Evaluation of leukopenia during sepsis as a marker of sepsis-defining organ dysfunction. PLoS One. (2021) 16:e0252206. doi: 10.1371/journal.pone.0252206, PMID: 34166406 PMC8224900

[31] Syed-Ahmed M Narayanan M. Immune Dysfunction and Risk of Infection in Chronic Kidney Disease. Adv Chronic Kidney Dis. (2019) 26:8–15. doi: 10.1053/j.ackd.2019.01.004, PMID: 30876622

[32] Crepin T Gaiffe E Courivaud C Roubiou C Laheurte C Moulin B. Pre-transplant end-stage renal disease-related immune risk profile in kidney transplant recipients predicts post-transplant infections. Transpl Infect Dis. (2016) 18:415–22. doi: 10.1111/tid.12534, PMID: 27027787

[33] Avagyan S Zon LI. Clonal hematopoiesis and inflammation - the perpetual cycle. Trends Cell Biol. (2023) 33:695–707. doi: 10.1016/j.tcb.2022.12.001, PMID: 36593155 PMC10310890

[34] Trowbridge JJ Starczynowski DT. Innate immune pathways and inflammation in hematopoietic aging, clonal hematopoiesis, and MDS. J Exp Med. (2021) 218:544. doi: 10.1084/jem.20201544, PMID: 34129017 PMC8210621

[35] Kishtagari A Corty RW Visconte V. Clonal hematopoiesis and autoimmunity. Semin Hematol. (2024) 61:3–8. doi: 10.1053/j.seminhematol.2024.01.012, PMID: 38423847

[36] Gurnari C Visconte V. From bone marrow failure syndromes to VEXAS: Disentangling clonal hematopoiesis, immune system, and molecular drivers. Leuk Res. (2023) 127:107038. doi: 10.1016/j.leukres.2023.107038, PMID: 36841022

[37] Jourdan M Tarte K Legouffe E Brochier J Rossi JF Klein B. Tumor necrosis factor is a survival and proliferation factor for human myeloma cells. Eur Cytokine Netw. (1999) 10:65–70. 10210775 PMC2025696

[38] Mantovani A Garlanda C. Inflammation and multiple myeloma: the Toll connection. Leukemia. (2006) 20:937–8. doi: 10.1038/sj.leu.2404229, PMID: 16721383

[39] Musolino C Allegra A Innao V Allegra AG Pioggia G Gangemi S. Inflammatory and Anti-Inflammatory Equilibrium, Proliferative and Antiproliferative Balance: The Role of Cytokines in Multiple Myeloma. Mediat Inflamm. (2017) 2017:1–24. doi: 10.1155/2017/1852517, PMID: 29089667 PMC5635476

[40] Rosean TR Tompkins VS Tricot G Holman CJ Olivier AK Zhan F. Preclinical validation of interleukin 6 as a therapeutic target in multiple myeloma. Immunol Res. (2014) 59:188–202. doi: 10.1007/s12026-014-8528-x, PMID: 24845460 PMC4209159

[41] Martinez-Lopez J Hernandez-Ibarburu G Alonso R Sanchez-Pina JM Zamanillo I Lopez-Muñoz N. Impact of COVID-19 in patients with multiple myeloma based on a global data network. Blood Cancer J. (2021) 11:198. doi: 10.1038/s41408-021-00588-z, PMID: 34893583 PMC8661359

[42] Carmichael J Seymour F McIlroy G Tayabali S Amerikanou R Feyler S. Delayed diagnosis resulting in increased disease burden in multiple myeloma: the legacy of the COVID-19 pandemic. Blood Cancer J. (2023) 13:38. doi: 10.1038/s41408-023-00795-w, PMID: 36922489 PMC10015143

[43] Ehsan H Britt A Voorhees PM Paul B Bhutani M Varga C. Retrospective Review of Outcomes of Multiple Myeloma (MM) Patients With COVID-19 Infection (Two-Center Study). Clin Lymphoma Myeloma Leuk. (2023) 23:273–8. doi: 10.1016/j.clml.2023.01.006, PMID: 36797155 PMC9847363

[44] Hansson E Forbes HJ Langan SM Smeeth L Bhaskaran K. Herpes zoster risk after 21 specific cancers: population-based case-control study. Br J Cancer. (2017) 116:1643–51. doi: 10.1038/bjc.2017.124, PMID: 28463961 PMC5518853

[45] Antwi-Amoabeng D Ulanja MB Beutler BD Reddy SV. Multiple myeloma remission following COVID-19: an observation in search of a mechanism (a case report). Pan Afr Med J. (2021) 39:117. doi: 10.11604/pamj.2021.39.117.30000, PMID: 34512853 PMC8396390

[46] Yamamoto T Aoyama Y. Immune reconstitution is the trigger of herpes zoster with lymphopenia and high neutrophil-to-lymphocyte ratio in a retrospective cohort study. Clin Exp Dermatol. (2024) 49:1372–8. doi: 10.1093/ced/llae176, PMID: 38723590

[47] Wang X Wen Y Xie X Liu Y Tan X Cai Q. Dysregulated hematopoiesis in bone marrow marks severe COVID-19. Cell Discov. (2021) 7:60. doi: 10.1038/s41421-021-00296-9, PMID: 34349096 PMC8335717

[48] Elahi S. Hematopoietic responses to SARS-CoV-2 infection. Cell Mol Life Sci. (2022) 79:187. doi: 10.1007/s00018-022-04220-6, PMID: 35284964 PMC8918078

