# Supplementary material for: Risk of hematologic malignancies following herpes zoster after COVID-19: a global cohort study
Source: Front Med (Lausanne). 2025 Sep 22;12:1651614. doi: 10.3389/fmed.2025.1651614 (PMC12497860; doi:10.3389/fmed.2025.1651614)
Supplement: Supplementary file 1 [file Table_1.DOCX]

Supplementary Material

| **Supplementary Table S1.** Baseline characteristics of COVID-19 patients aged 60–70 years before and after propensity score matching. | | | | | | | | |
| --- | --- | --- | --- | --- | --- | --- | --- | --- |
|  | Before Matching | | | | After Matching | | | |
| Characteristics^a^ | COVID-19  with HZ  (n=7404) | COVID-19  without HZ  (n=2,077,659) | *p* value | SMD | COVID-19  with HZ  (n=7404) | COVID-19  without HZ  (n=7404) | *p* value | SMD |
| Demographics | | | | | | | | |
| Age at Index,  mean ± SD | 60.8 ± 3.2 | 60.6 ± 3.2 | <0.01 | 0.06 | 60.8 ± 3.2 | 60.8 ± 3.2 | 0.60 | 0.01 |
| Female (%) | 60.9% | 51.6% | <0.01 | 0.19 | 60.9% | 59.5% | 0.09 | 0.03 |
| Male (%) | 36.6% | 46.5% | <0.01 | 0.22 | 36.6% | 37.9% | 0.10 | 0.03 |
| White (%) | 67.2% | 55.3% | <0.01 | 0.20 | 68.6% | 69.5% | 0.23 | 0.02 |
| Black or African American (%) | 12.3% | 14.3% | <0.01 | 0.06 | 12.2% | 12.1% | 0.78 | 0.01 |
| Asian (%) | 5.2% | 3.8% | <0.01 | 0.07 | 5.2% | 5.3% | 0.88 | <0.01 |
| Diagnosis (%) | | | | | | | | |
| Diabetes mellitus | 17.7% | 10.0% | <0.01 | 0.23 | 17.7% | 18.2% | 0.45 | 0.01 |
| Hypertensive diseases | 34.7% | 20.4% | <0.01 | 0.32 | 34.7% | 35.5% | 0.33 | 0.02 |
| Cerebrovascular diseases | 2.9% | 2.1% | <0.01 | 0.05 | 2.9% | 3.1% | 0.53 | 0.01 |
| Medication (%) | | | | | | | | |
| Antilipidemic agents | 21.1% | 13.1% | <0.01 | 0.21 | 21.1% | 22.0% | 0.17 | 0.02 |
| Diuretics | 14.5% | 8.7% | <0.01 | 0.18 | 14.5% | 14.0% | 0.40 | 0.01 |
| Beta blockers | 13.8% | 8.5% | <0.01 | 0.17 | 13.8% | 14.4% | 0.29 | 0.02 |
| Calcium channel blockers | 10.8% | 6.5% | <0.01 | 0.16 | 10.8% | 11.3% | 0.39 | 0.01 |
| Angiotensin II inhibitor | 8.1% | 5.1% | <0.01 | 0.12 | 8.1% | 8.3% | 0.74 | 0.01 |
| Blood glucose regulation agents | 15.1% | 8.9% | <0.01 | 0.19 | 15.1% | 15.5% | 0.51 | 0.01 |
| Laboratory^b^ (mean ± SD) | | | | | | | | |
| Creatinine, mg/dL | 1.1 ± 1.1 | 1.1 ± 1.6 | 0.76 | 0.01 | 1.1 ± 1.1 | 1.1 ± 1.1 | 0.92 | <0.01 |
| Urea, nitrogen | 18.3 ± 11.3 | 17.7 ± 10.3 | <0.01 | 0.05 | 18.3 ± 11.3 | 17.9 ± 10.0 | 0.15 | 0.03 |
| Lymphocytes, % | 25.6 ± 11.8 | 26.0 ± 11.3 | 0.12 | 0.03 | 25.6 ± 11.8 | 26.2 ± 11.4 | 0.07 | 0.05 |
| Glucose, mg/dL | 120.1 ± 54.0 | 118.5 ± 52.4 | 0.06 | 0.03 | 120.1 ± 54.0 | 121.7 ± 54.5 | 0.22 | 0.03 |
| Calcium, mg/dL | 9.3 ± 0.6 | 9.3 ± 0.6 | 0.80 | <0.01 | 9.3 ± 0.6 | 9.3 ± 0.6 | 0.94 | <0.01 |
| Phosphate, mg/dL | 3.6 ± 1.0 | 3.6 ± 1.1 | 0.29 | 0.05 | 3.6 ± 1.0 | 3.7 ± 1.0 | 0.30 | 0.06 |
| Hemoglobin, g/dL | 13.2 ± 2.0 | 13.4 ± 2.0 | <0.01 | 0.11 | 13.2 ± 2.0 | 13.2 ± 2.1 | 0.17 | 0.03 |
| Hematocrit, % | 39.5 ± 7.3 | 39.6 ± 8.5 | 0.56 | 0.01 | 39.5 ± 7.3 | 39.5 ± 7.7 | 0.96 | <0.01 |
| ALT, U/L | 27.3 ± 29.6 | 28.9 ± 52.0 | 0.08 | 0.04 | 27.3 ± 29.6 | 29.3 ± 68.3 | 0.13 | 0.04 |
| AST, U/L | 26.8 ± 25.1 | 29.5 ± 76.1 | 0.05 | 0.05 | 26.8 ± 25.1 | 28.3 ± 53.4 | 0.17 | 0.03 |
| Alkaline phosphatase, U/L | 91.0 ± 59.7 | 90.8 ± 65.4 | 0.85 | <0.01 | 91.0 ± 59.7 | 90.7 ± 55.2 | 0.82 | 0.01 |
| Albumin, g/dL | 4.1 ± 0.5 | 4.1 ± 0.5 | 0.28 | 0.02 | 4.1 ± 0.5 | 4.1 ± 0.5 | 0.92 | <0.01 |
| Protein, g/dL | 7.0 ± 0.7 | 7.1 ± 0.8 | 0.08 | 0.03 | 7.0 ± 0.7 | 7.0 ± 0.7 | 0.77 | 0.01 |
| Cholesterol, mg/dL | 184.1 ± 46.9 | 183.6 ± 46.1 | 0.61 | 0.01 | 184.1 ± 46.9 | 183.8 ± 47.7 | 0.84 | 0.01 |
| LDL, mg/dL | 103.7 ± 38.7 | 104.2 ± 38.0 | 0.60 | 0.01 | 103.7 ± 38.7 | 103.8 ± 39.7 | 0.96 | <0.01 |
| HDL, mg/dL | 49.9 ± 20.8 | 49.5 ± 20.7 | 0.44 | 0.02 | 49.9 ± 20.8 | 48.7 ± 21.5 | 0.09 | 0.05 |
| Triglyceride, mg/dL | 140.5 ± 97.3 | 141.4 ± 116.3 | 0.74 | 0.01 | 140.5 ± 97.3 | 144.5 ± 94.5 | 0.20 | 0.04 |
| HbA1c, % | 6.7 ± 1.8 | 6.6 ± 1.7 | 0.04 | 0.05 | 6.7 ± 1.8 | 6.7 ± 1.6 | 0.85 | 0.01 |
| PTH, pg/mL | 121.6 ± 159.6 | 126.2 ± 193.2 | 0.75 | 0.03 | 121.6 ± 159.6 | 121.6 ± 147.5 | 1.00 | <0.01 |
| Calcidiol, ng/mL | 37.2 ± 17.0 | 36.2 ± 17.8 | 0.30 | 0.05 | 37.2 ± 17.0 | 37.8 ± 18.1 | 0.61 | 0.04 |
| CRP, mg/L | 24.1 ± 53.0 | 29.1 ± 56.4 | 0.07 | 0.10 | 24.1 ± 53.0 | 29.1 ± 58.1 | 0.20 | 0.09 |
| Urate, mg/dL | 5.9 ± 2.0 | 5.9 ± 2.2 | 0.71 | 0.02 | 5.9 ± 2.0 | 5.9 ± 1.9 | 0.85 | 0.01 |
| Iron, µg/dL | 69.3 ± 42.8 | 72.3 ± 44.4 | 0.21 | 0.07 | 69.3 ± 42.8 | 69.2 ± 52.9 | 0.96 | <0.01 |
| Ferritin, ng/mL | 355.2 ± 687.9 | 345.8 ± 1118.8 | 0.86 | 0.01 | 355.2 ± 687.9 | 372.7 ± 742.3 | 0.72 | 0.02 |
| ESR, mm/h | 26.8 ± 24.9 | 27.6 ± 27.2 | 0.57 | 0.03 | 26.8 ± 24.9 | 28.2 ± 28.5 | 0.46 | 0.05 |

Abbreviations: COVID-19, coronavirus disease 2019; HZ, herpes zoster; SMD, standardized mean difference; ALT, alanine aminotransferase; AST, aspartate aminotransferase; LDL, low-density lipoprotein; HDL, high-density lipoprotein; HbA1c, hemoglobin A1c; PTH, parathyroid hormone; CRP, C-reactive protein; ESR, erythrocyte sedimentation rate; SD, standard deviation.

a. Data are presented as % unless otherwise indicated.

b. Laboratory data reflects the average result for each patient’s most recent test that occurred closest to their first document COVID-19 event.

**Supplementary Table S2.** Risk of Chronic Leukemia and Lymphoma Following Herpes Zoster Infection in Patients with COVID-19.

| **Outcome** | **Cohorts** | **Patients in cohort** | **Patients with outcome** | **Survival probability at end of time window** | **Hazard**  **ratio^a^** | **95%** **CI** | **Log-Rank**  **test**  ***p* value** |
| --- | --- | --- | --- | --- | --- | --- | --- |
| **Chronic leukemia** | COVID+HZ | 30,758 | 83 | 99.71% | 1.537 | (1.072, 2.204) | 0.018 |
|  | COVID-HZ | 30,903 | 46 | 99.80% |  |  |  |
| **Lymphoma** | COVID+HZ | 30,323 | 215 | 99.24% | 2.079 | (1.624, 2.662) | <0.001 |
|  | COVID-HZ | 30,761 | 89 | 99.62% |  |  |  |

Abbreviations: HZ, Herpes zoster; CI, confidence interval.

**Supplementary Table S3.** Healthcare Utilization Following COVID-19 With or Without Subsequent Herpes Zoster: Comparison of Outpatient and Inpatient Encounters.

| **Visit** | **Cohort** | **Patients in Cohort** | **Patients with Outcome** | **Mean Visits** | **Standard Deviation** | **p-value** |
| --- | --- | --- | --- | --- | --- | --- |
| **Ambulatory**  **(OPD)** | COVID+HZ | 31,015 | 28,770 | 43.94 | 52.521 | < 0.001 |
|  | COVID-HZ | 31,015 | 24,439 | 23.958 | 38.599 |  |
| **Inpatient**  **encounter** | COVID+HZ | 31,015 | 12,066 | 2.21 | 6.624 | < 0.001 |
|  | COVID-HZ | 31,015 | 8,883 | 1.208 | 4.064 |  |

Abbreviations: HZ, Herpes Zoster; OPD, Outpatient Department.
